# Gait velocity exhibits more than 50% diurnal variation in Rheumatoid Arthritis: the divign study

**DOI:** 10.1186/1757-1146-3-S1-P1

**Published:** 2010-12-20

**Authors:** Michael Backhouse, David Pickles, Hannah Mathieson, Lucy Edgson, Paul Emery, Howard Bird, Philip Helliwell, Anthony Redmond

**Affiliations:** 1 University of Leeds, Leeds, UK; 2 Leeds Teaching Hospitals NHS Trust, Leeds, UK

## Introduction

Gait velocity (GV) is a frequently used outcome measure in studies of the lower limb in RA as it is reliable & correlates with disease impact. Despite recent knowledge of marked circadian variation in inflammatory cytokine levels & upper limb function, little is known about within-day variation of gait in RA.

## Objectives

Describe patterns of diurnal variation of gait velocity in patients with RA

## Methods

Inpatients with RA walked at self-selected speed along an 8m GAITRite instrumented walkway (CIR Systems Inc, USA) 5 times during a single day; waking (0 hr), +1 hr, +3 hrs, +6, & +12 hrs. Walking aids were allowed as required.

## Results

Data were collected on 31 pts with RA (11 M, 20 F) median age of 67 (range 35 to 87), disease duration 10.5y (range 1 to 50), mean BMI 25.9 (std +/- 5.5 ). Median DAS28 5.39 (IQR 2.11) median HAQ 2.25 (IQR 0.79). The largest difference in GV between time points was the increase from 0hr to 1 hr (20.4% 95%CI 9.7 to 31.2) & 1hr to 3hrs (10.2% 95%CI 2.0 to 18.4). Between +3 & +6 was less (6% 95% CI -1.5 to 13.4) with a greater difference between 6 & 12 hrs (9.3% 95% CI 1.3 to 17.3) (Table 1).

**Table 1:** 

	0hr	+1hrs	+3hrs	+6hrs	+12hrs
Mean GV (cm/sec)	37.6 (+/-24.9)	43.8(+/- 28.7)	45.5 (+/- 26.9)	48.1 (+/- 29.0)	53.0 (+/- 32.2)
**Δ** from 0hr (95%CI)		20.4% (9.7 to 31.2)	32.9% (16.6 to 49.3)	37.8% (21.6 to 54.1)	54.1% (25.3 to 82.9)

## Conclusion

Patients with RA showed systematic diurnal variation in GV with a sharp increase in the first hour after waking and continued improvement throughout the day. Although the effect of morning stiffness is well recognised, this is the first time that its effect on gait has been quantified. These data have important implications for the interpretation of gait analysis and other measures of functional capacity: Repeat measures should be made at a similar time of day to exclude the effects of diurnal variation.

